# Pituitary Stalk Interruption Syndrome: A Case Report

**DOI:** 10.7759/cureus.30218

**Published:** 2022-10-12

**Authors:** Alishbah Ziad, Quratulain Khan, Hira Farooq, Anis Rehman, Kashif Siddique

**Affiliations:** 1 Radiology, Shaukat Khanum Memorial Cancer Hospital and Research Centre, Lahore, PAK

**Keywords:** mri, infundibulum, posterior pituitary, hormonal workup, pituitary stalk interruption syndrome

## Abstract

Pituitary stalk interruption syndrome is a congenital abnormality. The triad of this syndrome comprises a thin pituitary stalk, an ectopic posterior pituitary gland, and an absent or hypoplastic anterior pituitary gland. The patient typically presents with a spectrum of symptoms secondary to anterior pituitary hormonal deficiency. The etiology of this syndrome is not established but is likely due to a genetic mutation. The prognosis is good if the syndrome is diagnosed early and hormonal therapy is started promptly. Early diagnosis is crucial in preventing adverse effects on growth and development. The diagnosis of pituitary stalk interruption syndrome is based on magnetic resonance imaging (MRI) findings.

This study presents the case of a young girl who presented with complaints of short stature and amenorrhea and was diagnosed with pituitary stalk interruption syndrome following an MRI.

## Introduction

The reported incidence of pituitary stalk interruption syndrome is 0.5 per 100,000 live births [1]. The first-ever reported case was by Fujisawa et al. in 1987 after surgical resection of the pituitary stalk in a patient with idiopathic pituitary dwarfism [2]. The exact physiology of this syndrome is not fully understood. Multiple hypotheses exist, such as a perinatal anoxic breech presentation at the time of delivery, resulting in damage to the pituitary stalk. Undescended testes and other syndromic associations secondary to genetic mutations have also been described [3,4]. The triad of pituitary stalk interruption syndrome includes a thin pituitary stalk, aplasia, or hypoplasia of the anterior pituitary gland, or an ectopic posterior pituitary gland observed on magnetic resonance imaging (MRI). A multitude of additional symptoms may include short stature due to growth hormone deficiency and hypogonadism. Laboratory examinations show low levels of all the anterior pituitary hormones.

## Case presentation

This study discusses the case of a nine-year-old girl who first presented with short stature in 2014. Her insulin-like growth factor-1 (IGF-1) levels were decreased, and she was started on growth hormone (GH) supplements. No other tests from 2014 were available. Her growth velocity was 4 cm per year on GH supplements. She presented again in 2022 at the age of 16.5 years with a complaint of menstrual irregularities/amenorrhea. The patient had been delivered at term by cesarean section. There was a history of jaundice in the postnatal period for which she received phototherapy. The patient also had postnatal seizures for three days following birth. There was no other significant postnatal or family history. She did face some learning difficulties in school, however, no behavioral issues were seen. No history of weight gain was there.

The hormonal levels recorded at this visit showed decreased luteinizing hormone (LH), follicle-stimulating hormone (FSH), estradiol, free thyroxine (T4), and IGF-1 levels (Table 1). The patient did not have any records of lab tests performed with her in between.

**Table 1:** Hormonal levels, recorded in 2012 and 2022, showing decreased levels

	2012	2022	Normal range
Follicle-stimulating hormone	-	<0.12 IU/L	3–10 IU/L
Luteinizing hormone	-	<0.12 IU/L	2–8 IU/L
Estradiol	-	<10 ng/ml	30–40 ng/ml
Free thyroxine	-	0.50 ng/dl	0.9–1.4 ng/dl
Insulin-like growth factor-1	4.28 ng/dl	76.5 ng/ml	182–780 ng/dl

**Figure 1:**
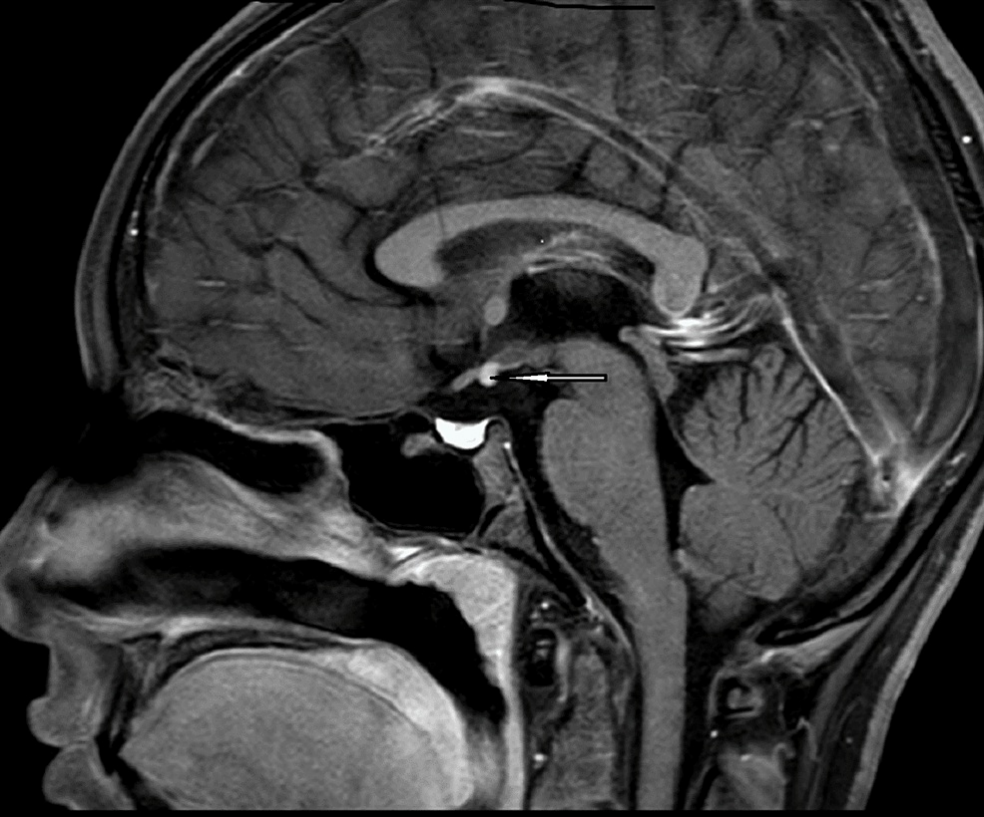
Sagittal post-contrast magnetic resonance imaging of the brain shows an absence of the posterior pituitary in the pituitary fossa The enhancing nodule, visible along the inferior aspect of the optic chiasma, is suggestive of an ectopic pituitary (depicted by a white arrow).

**Figure 2:**
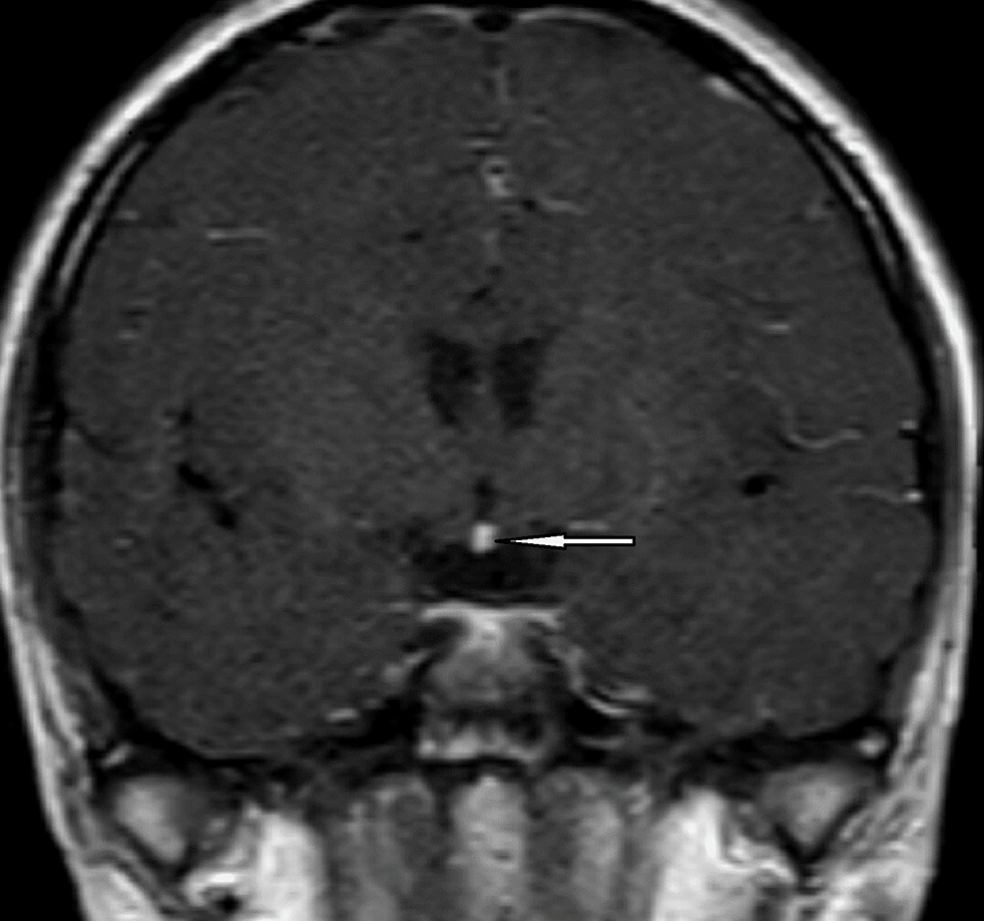
Coronal post-contrast magnetic resonance imaging of the brain shows an enhancing nodule at the inferior aspect of the optic chiasma (white arrow), suggestive of an ectopic posterior pituitary gland

**Figure 3:**
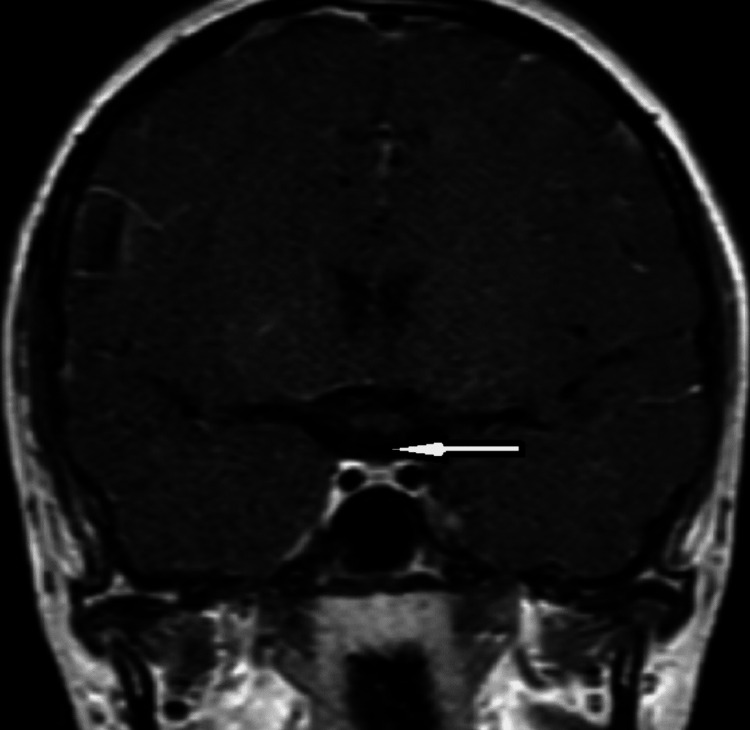
Coronal post-contrast magnetic resonance imaging of the brain shows that the pituitary stalk/infundibulum cannot be identified (as shown by the white arrow)

Ultrasonography of the pelvis showed a hypoplastic uterus and ovaries. An X-ray done of the hand elsewhere showed a bone age of 13-14 years. A brain MRI showed an ectopic posterior pituitary and an absent/hypoplastic pituitary stalk (Figures 1-3).

A diagnosis of pituitary stalk interruption syndrome was made, and the patient was started on hormone replacement therapy with regular follow-up visits with an endocrinologist.

## Discussion

The pituitary gland is an integral part of our endocrine system. It is pea-sized and sits below the hypothalamus within the sella turcica. The pituitary gland has two lobes, an anterior and posterior lobe, connected to the hypothalamus by the pituitary stalk, or infundibulum. Together, the hypothalamus and pituitary gland form the hypothalamus-pituitary complex. The pituitary gland secretes several hormones, including adrenocorticotropic hormone, LH, FSH, thyroid-stimulating hormone, and prolactin, secreted by the anterior pituitary, and anti-diuretic hormone and oxytocin, secreted by the posterior pituitary. The secretion of most of these hormones is governed by releasing hormones secreted by the hypothalamus, i.e. the gonadotropin hormone-releasing hormone, growth hormone-releasing hormone, corticotropin-releasing hormone, and thyrotropin-releasing hormone. Communication between the hypothalamus and the pituitary gland occurs through nerve pulses via the pituitary stalk/infundibulum, which comprises nerves and blood vessels.

During the first two years of life, the pituitary gland undergoes extensive morphological change. At birth, both the anterior and posterior pituitary show a high signal on T1-weighted MRI images [4,5]. At six months, the anterior pituitary loses this signal and becomes iso-intense. The posterior pituitary retains a high signal throughout life due to the presence of neurosecretory granules [6-8].

The pathophysiology of pituitary stalk interruption syndrome is not established. Few cases show a genetic mutation in the transcription factor HESX1 (3p21.2-p21.1) and in the LHX4 gene (1q25), however, in the majority of cases no genetic cause is found. Studies suggest antenatal origin as to the presence of familial forms and the association of PSIS with microphallus and congenital abnormalities, particularly of the eye.

Pituitary stalk interruption syndrome is a rare condition with variable presentation secondary to decreased levels of anterior pituitary hormones. This syndrome has a male predilection. Crucial steps in the condition’s management include an early diagnosis and prompt initiation of hormonal replacement therapy. The patient in this study case also presented with short stature and amenorrhea, secondary to decreased levels of anterior pituitary hormones. An MRI scan of the brain confirmed the classic findings of pituitary stalk interruption syndrome.

There are a large number of patients who presents to the endocrinology clinic every year with complaints secondary to hormonal insufficiency. The most critical issue faced by physicians in this scenario is to effectively diagnose and treat the underlying cause. As this condition requires a timely diagnosis for a good prognosis; otherwise, many complications, for example, seizures may develop. Rare conditions like ours must be kept under consideration. Our case report concludes the importance of MRI and hormonal profile in early diagnosis. It also confirms the available literature and emphasizes its importance in patient management.

## Conclusions

Pituitary stalk interruption syndrome is a rare congenital abnormality. Early diagnosis is crucial for preventing adverse effects on long-term growth and development. A diagnosis of pituitary stalk interruption syndrome is based on MRI findings.
